# Optimization of physicochemical and textural properties of pizza cheese fortified with soybean oil and carrot extract

**DOI:** 10.1002/fsn3.563

**Published:** 2018-01-05

**Authors:** Ehsan Motevalizadeh, Seyed Ali Mortazavi, Elnaz Milani, Moosa Al‐Reza Hooshmand‐Dalir

**Affiliations:** ^1^ Department of Food Science and Technology Azad Islamic University of Sabzevar Sabzevar Iran; ^2^ Iranian Academic Center for Education Culture and Research (ACECR) Mashhad Iran

**Keywords:** carrot extract, optimization, physicochemical and textural properties, pizza cheese

## Abstract

Response surface methodology (RSM) was used to optimize pizza cheese containing carrot extract. The effects of two important independent variables including soybean oil (5%–20%) and carrot extract (5%–20%) were studied on physicochemical and textural properties of pizza cheese containing carrot extract. According to the results, RSM was successfully used for optimizing formulation of pizza cheese containing carrot juice. Results of this study revealed that oil (A), carrot (B), AB, square term of carrot (B^2^), B, AB, square term of oil (A^2^), B^2^, AB, AB, A^2^B, A^2^, A^2^, A, A^2^, A^2^, AB, and AB ^2^ had the most effect on moisture, acidity, stretch, L*, a*, b*, hardness, meltability, springiness, peroxide value (PV), cohesiveness, chewiness, gumminess, fracture force, adhesiveness force, stiffness, flavor, and overall acceptability, respectively. A formulation upon 20% oil and 10.88% carrot extract was found as the optimal formulation for pizza cheese containing carrot extract. At the optimal formulation, PV, L*, a*, b*, meltability, stretch, cohesiveness, springiness, gumminess, chewiness, adhesive force, flavor, texture, and overall acceptability at the optimum formulation were measured 2.23, 82.51, −3.69, 18.05, 17.86, 85.61, 0.41, 7.874, 23.7, 0.27, 0.61, 3.50, 3.95, and 3.65, respectively.

## 1 INTRODUCTION

Food industry is seeking for approaches to improve organoleptic characteristics and shelf life of products, such as adding some natural preservatives. On the other hand, food consumers are seeking natural additives and/or traditional medicines in foods instead of synthetic types. Plants represent a large source of natural bioactive substances that may show health‐promotion effect (Salminen, Lehtonen, Suuronen, Kaarniranta, & Huuskonen, 2008, Azizpour, M., Mohebbi, M., Yolmeh, M., Abbasi, E., & Sangatash, M. M. (2017)). Advantage of medicinal plants has been known for centuries, and therapeutic benefit of several herbal species has been widely described and these plants might act as an alternative treatment of infectious diseases (Natarajan, Venugopal, & Menon, 2003). Giving the World Health Organization (WHO), medicinal plants would be the best source for acquiring a variety of drugs (Lewis & Ausubel, 2006). This evidence gives to support and quantify the importance of screening natural products.

The carrot (*Daucus carota* subsp. *sativus*) is a root vegetable, which is usually orange in color, though purple, black, red, white, and yellow varieties exist. Carrot has a brittle texture when fresh. Taproot is the most commonly eaten part of a carrot, notwithstanding the greens are occasionally eaten as well. It is a tamed form of the wild carrot *Daucus carota*, native to Europe and southwestern Asia. The domestic carrot has been selectively bred for its greatly enlarged and more palatable, less woody‐textured edible taproot (Bishayee, Sarkar, & Chatterjee, 1995). The Food and Agriculture Organization (FAO) reports that world production of carrots and turnips for the calendar year 2011 was about 35.658 million tons. Almost half were grown in China. Carrots are widely used in many cuisines, especially in the preparation of salads. Carrot extract is rich in vitamins A, E, and β‐carotene. It is an anti‐inflammatory and soothing to chapped and uncomfortable skin (Mahran et al., 1991). Carrots are widely used to calm the nervous system and its scraped root is used as a local stimulant for inactive ulcers.

Pizza cheese is similar to low‐moisture, part‐skim Mozzarella cheese in functional and organoleptic properties, but it is a nonpasta filata, stirred‐curd cheese manufactured, using *Lactococcus lactis* ssp. *cremoris* and *Lactococcus lactis* ssp. *lactis* (Chen & Johnson, 2001). Pizza cheese is usually produced from milk with a CN:fat ratio of about 1.0–1.05, and milk can be standardized by cream removal or addition of condensed and/or ultrafiltration (UF) milk and/or nonfat dry milk (NDM).

Response surface methodology (RSM) is a suitable statistical technique to optimize processes and formulation, which recently have been widespread used in the food industry to optimize different processes (Yolmeh & Jafari, 2017). RSM can decrease the number of experiments and properly shows the interactive effect of independent variables on responses, which are used to optimize cheese formulations (Farbod et al., 2014; Jooyandeh, Goudarzi, Rostamabadi, & Hojjati, 2017; and Khetra, Kanawjia, & Puri, 2016).

The lactic method is the most important production method to produce pizza cheese in the world. In addition, the citric and Cheese blend methods are used to produce this cheese. Uncertainty of production formula, lack of knowledge about the role and impact of the ingredients, low quality raw materials, production traditional method, optimization of the process, improper packaging, and lack of standard methods to examine the physical properties of the product are the difficulties of producing pizza cheese (Law & Tamime, 2011).

The objectives of this study were as follows: (1) development of pizza cheese containing carrot extract; (2) measurement of physicochemical and textural properties; (3) determination of an optimum formulation for this pizza cheese through RSM.

## 2 MATERIALS AND METHODS

### 2.1 Materials

Primary cheese was purchased from Chenaran dairy Industry Co. Mashhad in the Khorasan‐e‐Razavi province. Soybean oil was procured from Behpak Co. (Behshahr, Iran). All chemicals and solvents used in this study were of analytical reagent grade and prepared by Merck (Germany) and Sigma‐Aldrich (USA) Chemical Companies.

### 2.2 Carrot extract

The extract was prepared from carrot, using Bush extracts (CNCJ03 model). The extract was then concentrated from 8.6 to 34.5 Bx at 20°C and 85 mmHg, using a rotary evaporator (Heidolph Co., Germany Laborota 4003 model).

### 2.3 Preparation of pizza cheese

Pizza cheese was produced using the method described by Farkye, N. Y., & Yim, B. (2003) with some modification. Briefly, primary cheese was poured into the cooking pot, and then the temperature was increased up to 60°C to separate remaining whey. After separating whey, half of the other ingredients containing soybean oil, carrot extract, cream, salt, sodium nitrate, and sodium phosphate were added to the cheese at 80°C. After 15 min and good mixing the above ingredients with primary cheese, other ingredients were added to cheese. After 15 min, the temperature was decreased to 75°C. The prepared pizza cheeses were packed into polypropylene bags and stored in a refrigerator at 4°C.

### 2.4 Chemical analysis

#### 2.4.1 Moisture

Pizza cheese samples were dried in the vacuum oven and moisture content was measured, using the method described by Daniela, Gustavo, and Barbosa (2012).

#### 2.4.2 Acidity

Acidity of pizza cheese was reported as oleic acid and measured by the following formula (AOAC, 1990):

$$\text{Acidity}\left(\%\right)=\frac{V\times N\times 28.2}{w}$$

*V*: volume of used NaOH (mL); *W*: weight of the sample (g); *N*: normality of the used NaOH.

### 2.5 Color analysis

The color properties of pizza cheese samples (L*, a*, and b* values) were evaluated, using the method developed by Zahedi and Mazaheri‐Tehrani (2012).

### 2.6 Peroxide value (PV)

The spectrophotometric method of the International Dairy Federation as described by Azizpour, Najafzadeh, Yolmeh, and Sangatash (2017) was used to determine PV. The PV of pizza cheese samples were calculated, using the following formula:

$$\text{Peroxide value}=\frac{V\times N\times 1000}{W}$$

where *V*,* N*,* W* represent volume of used sodium thiosulfate (ml), normality of sodium, and weight of the sample (g), respectively.

### 2.7 Textural analysis

Melting, stretch‐ability, and texture profile analyses were carried out using the method described by Zisu et al. (2007). The texture measurements were performed, using TA Plus texture analyzer (AMETEK, UK) connected to a computer programmed with Nexygen 3 software. A flat cylindrical probe (30 mm in diameter) was attached to a 0.5 kg compression load, while the target value was set at 20 mm with the speed of 1 mm/s. The Samples (50 g) were placed in a cylindrical vessel, (44 mm internal diameter × 70 mm deep). The Probe was set to penetrate the samples at a depth of 0.2 cm.

Texture profile analysis (TPA) was based on the calculation of instrumental hardness (the peak force estimated during the first compression cycle), instrumental cohesiveness (the ratio of the positive force area during the second compression to that during the first compression), instrumental adhesiveness (the negative force area of the first compression cycle), springiness (the height or deformation food that goes back to the previous state during the end of the first compression cycle and starting the second cycle), instrumental gumminess (hardness × cohesiveness), and instrumental chewiness (gumminess × springiness).

### 2.8 Sensory evaluation

The sensory properties of the pizza cheese samples, namely flavor, stiffness, and overall acceptability were evaluated by 20‐member trained panelists (10 females and 10 males) took part in the descriptive analysis. The evaluation was done in a climate‐controlled sensory evaluation laboratory. The panelists washed their palates between samples with water. The samples were served at room temperature (24 ± 1°C) and analyses were performed under normal lighting conditions on a 5‐point hedonic scale (from dislike extremely = 1 to like extremely = 5) (Sun & Brosnan, 2003).

### 2.9 Experimental design

A three level, two variable box– behnken design was employed to optimize with respect to two independent variables oil (%) and carrot (%). The independent variables and their levels are shown in Table 1. Regression analysis was done on the data of dependent variables. In order to analyze the obtained result design, expert software version 8 was used.

**Table 1:** Uncoded and coded levels of the independent variables

Independent variables	Symbol	Coded levels		
		−1	0	1
Oil (%)	X_1_	5	12.5	20
Carrot (%)	X_2_	5	12.5	20

### 2.10 Statistical analysis

The multiple regression equation was employed to fit the second‐order polynomial equation based on the observed results as follows:

$$Y=\beta_{k0}+\sum_{i=1}^{4}\beta_{\mathrm{ki}}x_{i}+\sum_{i=1}^{4}\beta_{\mathrm{kii}}x_{i}^{2}+\sum_{i<j=2}^{4}\beta_{\mathrm{kij}}x_{i}x_{j}$$

where *Y* represents the predicted response; β_*k*0_, β_*ki*_, β_*kii*_, and β_*kij*_ represent regression coefficients; and *x*_*i*_, *x*_*j*_ are the coded independent factors. The models were compared based on *R* ^2^, *R* ^2^‐adj, and *R* ^2^‐pred. *R* ^2^ values closer to 1, indicate that the model is more accurate (Ghorbannezhad, Bay, Yolmeh, Yadollahi, & Moghadam, 2016). After selecting the most accurate model, the analysis of variance (ANOVA) was used to investigate the statistical significance of the regression coefficients by Dunkan's test at 95% confidence level. The interactive effects of the factors were studied, using surface plots derived from the selected model (Yolmeh & Sadeghi Mahoonak, 2016).

The aim of the optimizing formulation of pizza cheese was to maximize the meltability, stretch, L*, b*, cohesiveness, springiness, flavor, texture, overall acceptability (OA), and minimize gumminess, chewiness, Adhesive Force, and PV with the same weight (*w* = 1). The credibility of the optimum formulation was diagnosed by the desirability values of the responses that range from 0 to 1. The closer values of desirability to 1 showed the more credible and desirable optimal formulation.

## 3 RESULTS AND DISCUSSION

### 3.1 Fitting the response surface models

According to the used design, 13 experiments were performed thrice and the obtained results are shown in Table 2.

**Table 2:** The formulation and the experimental data for the responses

Formulation	X_1_	X_2_	Moisture	Acidity	Stretch	L*	a*	b*	Hardness	Meltability	Springiness	PV
1	5	20	44.05	0.15	325	83.18	−3.32	18.51	40.08	18	11.07009	2
2	20	12.5	41.73	0.13	135	85.14	−3.71	17.96	58.4	17	8.415037	2.3
3	12.5	12.5	43.06	0.13	67	84.45	−3.47	17.54	59.5	19	8.006708	2.5
4	12.5	20	40.92	0.14	159	81.85	−3.34	19.54	60.66	15	7.413645	2.2
5	20	5	41.52	0.12	130	81.89	−3.6	18.6	45.42	16	7.267163	2.1
6	12.5	12.5	43.06	0.13	170	84.45	−3.47	17.54	59.5	19	8.086868	2.5
7	12.5	5	43.84	0.14	102	85.03	−3.78	17.5	59.6	18	7.950548	2.7
8	20	20	41.03	0.15	76	84.96	−3.69	17.54	32.04	16	5.521496	2.6
9	12.5	12.5	43.06	0.13	76	84.45	−3.47	17.54	59.5	19	7.515004	2.5
10	12.5	12.5	43.06	0.14	128	84.45	−3.47	17.54	59.5	19	8.121508	2.5
11	5	12.5	41.88	0.15	277	85.83	−3.5	16.82	44.97	22	7.778051	2.8
12	5	5	44.15	0.13	61	84.62	−3.76	16.96	44.24	17	7.654543	2.4
13	12.5	12.5	43.06	0.13	47	84.45	−3.47	17.54	59.5	19	8.400719	2.5

Formulation	X_1_	X_2_	Cohesiveness	Chewiness	Gumminess	Fracture force	Adhesiveness force	Stiffness	Flavor	Overall acceptability
1	20	65	0.4964	0.2203	19.9	38.1021	0.734579	7565.862	2.87	3.4
2	10	65	0.4855	0.2386	26.97	0.03602	0.746544	8854.256	4	3.9
3	30	65	0.4504	0.2156	26.96	64.6688	0.8695	10390.53	3.02	3.28
4	30	50	0.4333	0.2431	27	0.07595	0.797106	11548.54	3	3
5	20	50	0.3187	0.2052	17.47	0.11357	0.200782	6774.959	2.37	3
6	20	80	0.4504	0.2156	26.96	0.09495	0.8695	11102.27	3.02	3.28
7	10	50	0.48	0.2274	27.9	0.0756	0.894655	9354.848	3.5	2.82
8	20	80	0.54	0.1955	17.3	30.9321	0.886513	5301.935	3.37	3.7
9	20	50	0.4504	0.2156	26.96	0.10665	0.8695	9072.86	3.02	3.28
10	10	10	0.4504	0.2156	26.96	0.09499	0.8695	8346.976	3.02	3.28
11	10	65	0.4551	0.1592	20.46	44.6990	0.41717	6573.294	2.87	2.7
12	30	80	0.4472	0.1514	19.79	42.3159	0.282531	7847.603	3.87	3.6
13	30	65	0.4504	0.2156	26.96	0.05698	0.8695	6448.026	3.02	3.28

PV, peroxide value.

The values of *R* ^2^, *R* ^2^‐adj, and *R* ^2^‐pred revealed that the linear model was more adequate than other models for moisture and fracture force values of pizza cheese samples; however for acidity, stretch, a*, PV, cohesiveness, flavor, and overall acceptability, 2FI model was suitable. The quadratic model had more accuracy on L*, b*, hardness, chewiness, gumminess, adhesiveness force, and stiffness of pizza cheese containing carrot extract. However, for meltability and springiness, the cubic model was more adequate (Table 3). The selected models are as follows:

Moisture=42.65−0.97A−0.59B

Acidity=0.67−0.055A−0.015B+0.055AB

$$\begin{array}{cc} \text{PV} & =2.53-0.25A-0.25B+0.23\text{AB}-0.059A^{2}-0.16B^{2}+0.27A^{2}B+\\ & 0.32{\text{AB}}^{2} \end{array}$$

$$\begin{array}{cc} L^{*} & =84.56-0.34A-1.59B+1.13\text{AB}+0.64A^{2}-1.40B^{2}+2.00A^{2}B\\ & +0.11{\text{AB}}^{2} \end{array}$$

$$a^{*}=-3.54-0.07A+0.13B-0.13\text{AB}$$

$$b^{*}=17.60+0.30A+0.42B-0.65\text{AB}-0.38A^{2}+0.75B^{2}$$

$$\begin{array}{cc} \text{Cohesiveness} & =0.45+0.015A-0.023B+0.043\text{AB}+0.007A^{2}-0.006B^{2}\\ & +0.091A^{2}B-0.036{\text{AB}}^{2} \end{array}$$

$$\text{Hardness}=61.14+1.1A-2.75B-2.31\text{AB}-13.55A^{2}-5.1B^{2}$$

$$\text{Gumminess}=27.73+0.26A-0.16B-0.07\text{AB}-5.94A^{2}-2.21B^{2}$$

$$\text{Chewiness}=0.22+0.018A+0.012B-0.020\text{AB}-0.029A^{2}+0.007B^{2}$$

$$\text{Adhesiveness force}=0.87+0.067A-0.17B+0.058\text{AB}-0.30A^{2}-0.039B^{2}$$

$$\begin{array}{cc} \text{Springiness}= & 8.01+0.32A-0.27B-1.29\text{AB}+0.13A^{2}-0.28B^{2}\\ & +0.69A^{2}B-1.80{\text{AB}}^{2} \end{array}$$

Stretch=134.85−53.67A+44.50B−79.50AB

$$\text{Meltability}=19.03-1.33A-0.33B-0.25\text{AB}+0.38A^{2}-2.62B^{2}$$

$$\begin{array}{cc} \text{Flavor} & =3.1+0.56A-0.25B+0.50\text{AB}+0.15A^{2}-0.033B^{2}+0.23A^{2}B\\ & -0.82{\text{AB}}^{2} \end{array}$$

Texture=3.27+0.71A−0.38B

$$\begin{array}{cc} \text{OA} & =3.21+0.6A+0.09B+0.23\text{AB}+0.26A^{2}-0.13B^{2}\\ & +0.035A^{2}B-0.68{\text{AB}}^{2} \end{array}$$

**Table 3:** The statistics of the four fitted models

Models	Statistics	Responses									
		Moisture	Acidity	Stretch	L*	a*	b*	Hardness	Meltability	Springiness	PV
Linear	*R* ^2^	71.76	46.09	34.70	4.94	48.44	25.39	4.58	28.23	28.52	15.31
	*R* ^2^‐adj	62.11	35.31	21.64	−14.08	38.13	10.46	−14.50	14.00	14.22	2.63
	*R* ^2^‐pred	46.75	−31.62	−38.06	−38.12	11.23	5.75	−38.40	−44.07	−28.13	−5.58
2FI	*R* ^2^	72.01	74.69	64.79	34.51	79.94	52.19	6.44	28.96	67.32	57.57
	*R* ^2^‐adj	56.02	66.25	53.05	12.68	65.25	36.25	−24.75	5.28	56.43	42.76
	*R* ^2^‐pred	−13.80	37.50	59.84	−43.55	52.11	14.40	−51.27	−72.11	22.80	19.61
Quadratic	*R* ^2^	76.99	74.94	79.18	66.32	87.54	77.32	80.42	79.48	68.62	55.94
	*R* ^2^‐adj	36.26	57.04	64.31	42.26	78.63	61.12	66.43	64.82	46.21	24.46
	*R* ^2^‐pred	−80.54	−94.69	15.96	−34.00	5.05	39.45	35.38	28.61	−98.18	−29.91
Cubic	*R* ^2^	88.62	82.03	81.17	97.35	97.37	97.32	91.51	99.89	97.52	94.44
	*R* ^2^‐adj	72.70	56.87	54.80	93.64	93.69	94.31	79.63	99.74	94.05	86.65
	*R* ^2^‐pred	−92.97	−98.78	−58.53	−30.77	−32.69	−10.87	−96.39	87.48	89.08	−46.34

Models	Statistics	Responses							
		Cohesiveness	Chewiness	Gumminess	Fracture force	Adhesiveness force	Stiffness	Flavor	Overall acceptability
Linear	*R* ^2^	29.77	33.19	33.19	78.08	28.38	25.75	15.67	25.35
	*R* ^2^‐adj	15.72	19.83	19.83	69.45	14.05	17.35	9.58	12.58
	*R* ^2^‐pred	−67.12	5.49	14.99	57.34	4.42	4.75	3.57	4.07
2FI	*R* ^2^	64.69	50.91	50.91	38.72	30.25	40.92	79.16	80.01
	*R* ^2^‐adj	49.59	34.55	34.55	24.97	17.00	32.35	18.88	66.68
	*R* ^2^‐pred	20.79	24.80	4.80	15.54	5.27	3.80	55.47	49.65
Quadratic	*R* ^2^	23.42	78.03	78.03	53.07	75.64	82.01	85.32	43.67
	*R* ^2^‐adj	−69.54	73.34	62.34	32.41	58.25	74.42	54.47	33.43
	*R* ^2^‐pred	0.13	59.43	49.86	11.74	47.65	59.35	15.47	12.57
Cubic	*R* ^2^	98.43	94.88	94.88	45.98	99.90	88.35	89.65	87.87
	*R* ^2^‐adj	96.24	87.70	87.70	19.65	99.77	61.35	65.56	70.64
	*R* ^2^‐pred	−82.02	−27.64	−24.31	−20.42	88.65	15.55	−5.35	−11.57

PV, peroxide value.

In addition, the lack‐of‐fit of the selected models were insignificant (*p *> .05), which shows a high suitability of the models to predict the dependent variables.

The analysis of variance (ANOVA) was used to appraise the significance of the quadratic polynomial models. For each terms in the models, a small *p*‐value and a large *F*‐value shows a more significant effect on the response (Yolmeh, Khomeiri, Ghorbani, Ghaemi, & Ramezanpour, 2017). Thus, oil had the most effect on moisture, stretch, fracture force, and overall acceptability of pizza cheese samples; whereas, carrot had the most effect on acidity and a* of pizza cheese containing carrot. The quadratic term of oil (A^2^) had the most effect on hardness, chewiness, gumminess, adhesiveness force, and stiffness of pizza cheese samples. The interaction between oil and carrot (AB) had the most effect on L*, springiness, PV, flavor of the samples. The interaction between the quadratic term oil and carrot, and the quadratic term of carrot (B^2^) had the most effect on cohesiveness and L*, respectively (Table 4).

**Table 4:** ANOVA of the models for the responses

Source	Moisture				Acidity				Stretch				L*			
	DF	Mean of squares (MS)	*F*	*p*	DF	MS	*F*	*p*	DF	MS	*F*	*p*	DF	MS	*F*	*p*
Model	1	5.61	6.71	.025	2	0.0002	5.24	.027	3	18147.7	5.52	.02	7	2.39	26.26	.0012
Oil (A)	1	5.61	6.71	.025	1	0.0001	2.77	.127	1	17280.6	5.26	.047	1	0.24	2.61	.1668
Carrot (B)	1	4.31	5.61	.035	1	0.0004	7.7	.019	1	11881.5	3.61	.09	1	5.06	55.53	.0007
AB	—	—	—	—	—	—	—	—	1	25281	7.69	.021	1	5.09	55.85	.0007
A^2^	—	—	—	—	—	—	—	—	—	—	—	—	1	1.14	12.53	.0166
B^2^	—	—	—	—	—	—	—	—	—	—	—	—	1	5.43	59.64	.0006
A^2^B	—	—	—	—	—	—	—	—	—	—	—	—	1	5.32	58.43	.0006
AB^2^	—	—	—	—	—	—	—	—	—	—	—	—	1	0.015	0.17	.6978
A^3^	—	—	—	—	—	—	—	—	—	—	—	—	0	—	—	—
B^3^	—	—	—	—	—	—	—	—	—	—	—	—	0	—	—	—
Residual error	11	0.84	—	—	10	0.00005	—	—	9	3287.8	—	—	5	0.091		
Lack of fit	7	1.31	4.87	.075	6	0.00007	3.84	.107	5	3892.2	1.54	.35	1	0.46	17.87	.097
Pure error	4	0	—	—	4	0.00002	—	—	4	2532.3	—	—	4	0	—	—
Total	12	—	—	—	12	—	—	—	12	—	—	—	12	—	—	—

Source	a*				b*				Hardness				Meltability			
	DF	Mean of squares (MS)	*F*	*p*	DF	MS	*F*	*p*	DF	MS	*F*	*p*	DF	MS	*F*	*p*
Model	3	0.068	8.51	.005	5	0.98	4.77	.032	5	184.1	5.75	.02	5	6.36	5.42	.023
Oil (A)	1	0.029	3.69	.087	1	0.55	2.65	.147	1	7.19	0.22	.65	1	10.67	9.09	.019
Carrot (B)	1	0.1	13.04	.005	1	1.07	5.18	.057	1	45.27	1.41	.273	1	0.67	0.57	.475
AB	1	0.07	8.8	.016	1	1.7	8.27	.024	1	21.25	0.66	.442	1	0.25	0.21	.658
A^2^	—	—	—	—	1	0.39	1.89	.211	1	506.7	15.83	.005	1	0.4	0.34	.579
B^2^	—	—	—	—	1	1.57	7.64	.028	1	71.85	2.24	.178	1	18.97	16.17	.005
A^2^B	—	—	—	—	—	—	—	—	—	—	—	—	—	—	—	—
AB^2^	—	—	—	—	—	—	—	—	—	—	—	—	—	—	—	—
A^3^	—	—	—	—	—	—	—	—	—	—	—	—	—	—	—	—
B^3^	—	—	—	—	—	—	—	—	—	—	—	—	—	—	—	—
Residual error	9	0.008	—	—	7	0.21	—	—	7	32.02	—	—	7	1.17	—	—
Lack of fit	5	0.014	2.91	.065	3	0.48	5.03	.064	3	74.71	5.07	.055	3	2.74	4.83	.067
Pure error	4	0	—	—	4	0	—	—	4	0	—	—	4	0	—	—
Total	12	—	—	—	12	—	—	—	12	—	—	—	12	—	—	—

Source	Springiness				Chewiness				Cohesiveness				PV			
	DF	Mean of squares (MS)	*F*	*p*	DF	MS	*F*	*p*	DF	MS	*F*	*p*	DF	MS	*F*	*p*
Model	7	2.39	28.08	.001	7	0.085	12.13	.007	7	0.004	44.89	.0003	5	0.001	4.97	.029
Oil (A)	1	0.2	2.38	.183	1	0.13	17.9	.008	1	0.0004	4.97	.0763	1	0.002	7.16	.031
Carrot (B)	1	0.14	1.69	.25	1	0.12	17.9	.008	1	0.001	11.72	.0188	1	0.001	3.42	.107
AB	1	6.66	78.23	.0003	1	0.2	29	.003	1	0.007	79.57	.0003	1	0.001	5.65	.049
A^2^	1	0.047	0.56	.488	1	0.009	1.36	.296	1	0.0001	1.61	.2606	1	0.002	8.48	.022
B^2^	1	0.22	2.6	.167	1	0.069	9.95	.025	1	0.0001	1.17	.328	1	0.0001	0.55	.483
A^2^B	1	0.63	7.37	.042	1	0.1	14.44	.012	1	0.011	118.58	.0001	—	0.0002	—	—
AB^2^	1	4.33	50.89	.001	1	0.14	20.17	.006	1	0.001	19.01	.0073	—	0.0006	—	—
A^3^	0	—	—	—	0	—	—	—	0	—	—	—	—	0	—	—
B^3^	0	—	—	—	0	—	—	—	0	—	—	—	—	—	—	—
Residual error	5	0.085	—	—	5	0.007	—	—	5	0.0001	—		7	—	—	—
Lack of fit	1	0.011	0.11	.762	1	0.035	5.74	.067	1	0.0004	5.08	.0674	3	—	3.5	.102
Pure error	4	0.1	—	—	4	0	—	—	4	0	—	—	4	—	—	—
Total	12	—	—	—	—	—	—	—	12	—	—	—	12	—	—	—

Source	Gumminess				Fracture force				Adhesiveness force				Stiffness			
	DF	Mean of squares (MS)	*F*	*p*	DF	MS	*F*	*p*	DF	MS	*F*	*p*	DF	MS	*F*	*p*
Model	5	32.56	4.69	.038	2	795.87	1.59	.252	5	0.11	4.35	.04	7	0.000003	1.13	.461
Oil (A)	1	0.42	0.061	.812	1	1473.77	2.94	.117	1	0.027	1.05	.34	1	0.000002	0.78	.417
Carrot (B)	1	0.15	0.022	.886	1	117.97	0.24	.638	1	0.18	7.11	.032	1	0.000002	0.72	.434
AB	1	0.02	0.002	.96	—	—	—	—	1	0.014	0.54	.487	1	0.00003	0.11	.757
A^2^	1	97.57	14.05	.007	—	—	—	—	1	0.25	10	.016	1	2E−`	4.89	.078
B^2^	1	13.47	1.94	.206	—	—	—	—	1	0.004	0.17	.695	1	0.00002	0.078	.791
A^2^B	—	—	—	—	—	—	—	—	—	—	—	—	1	0.000003	0.94	.376
AB^2^	—	—	—	—	—	—	—	—	—	—	—	—	1	0.000005	1.56	.267
A^3^	—	—	—	—	—	—	—	—	—	—	—	—	0	—	—	—
B^3^	—	—	—	—	—	—	—	—	—	—	—	—	0	—	—	—
Residual error	7	6.95	—	—	10	501.79	—	—	7	0.025	—	—	5	0.000003	—	—
Lack of fit	3	16.21	4.17	.081	6	280.23	0.34	.887	3	0.059	2.87	.107	1	0.000003	1.03	.368
Pure error	4	0	—	—	4	834.13	—	—	4	0	—	—	4	0.000003	—	—
Total	12		—	—	12	—	—	—	12	—	—	—	12	—	—	—

Source	Flavor				Overall acceptability			
	DF	Mean of squares (MS)	*F*	*p*	DF	MS	*F*	*p*
Model	7	0.3	7.25	.022	7	0.17	5.12	.045
Oil (A)	1	0.64	15.58	.011	1	0.72	21.3	.006
Carrot (B)	1	0.13	3.05	.141	1	0.016	0.48	.52
AB	1	1	24.41	.004	1	0.2	5.99	.058
A^2^	1	0.064	1.54	.267	1	0.19	5.48	.066
B^2^	1	0.003	0.074	.796	1	0.047	1.4	.289
A^2^B	1	0.083	2.03	.213	1	0.001	0.048	.834
AB^2^	1	0.89	21.62	.005	1	0.61	17.98	.008
A^3^	0	—	—	—	0	—	—	—
B^3^	0	—	—	—	0	—	—	—
Residual error	5	0.041	—	—	5	0.034	—	—
Lack of fit	1	0.2	4.52	.097	1	0.17	5.37	.062
Pure error	4	0	—	—	4	0	—	—
Total	12	—	—	—	12	—	—	—

PV, peroxide value.

### 3.2 Effects of independent variables on the responses

#### 3.2.1 Moisture

**Figure 1:**
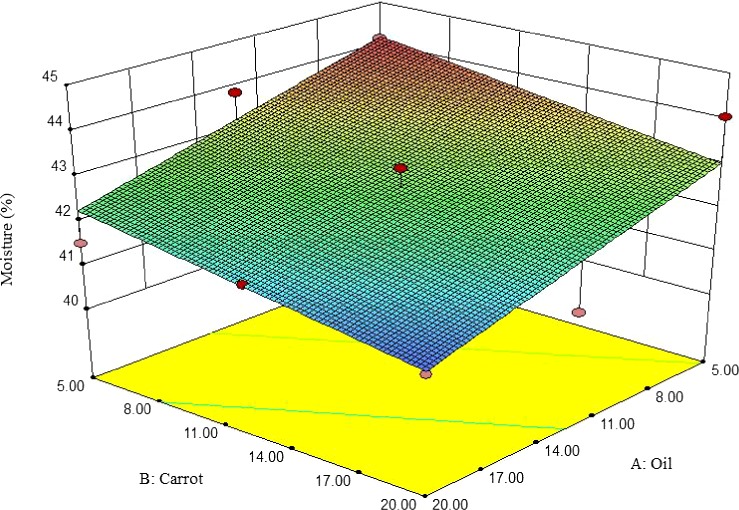
The interactive effect of soybean oil and carrot extract on moisture content of pizza cheese

The moisture content of pizza cheese containing carrot extract was decreased by adding oil and the extract so that the lowest moisture content (41.09) was observed at 20% of oil and carrot extract (Figure 1). Similarly, Ghanbari, Khosroshahi, Mortazavi, and Tavakolipour (2012) reported the same findings for Iranian low‐fat white cheese. Romeih, Michaelidou, Biliaderis, and Zerfiridis (2002) reported that the water holding capacity (WHC) of the matrix of casein was increased by decreasing oil, which leads to an increase in moisture content.

#### 3.2.2 Acidity

**Figure 2:**
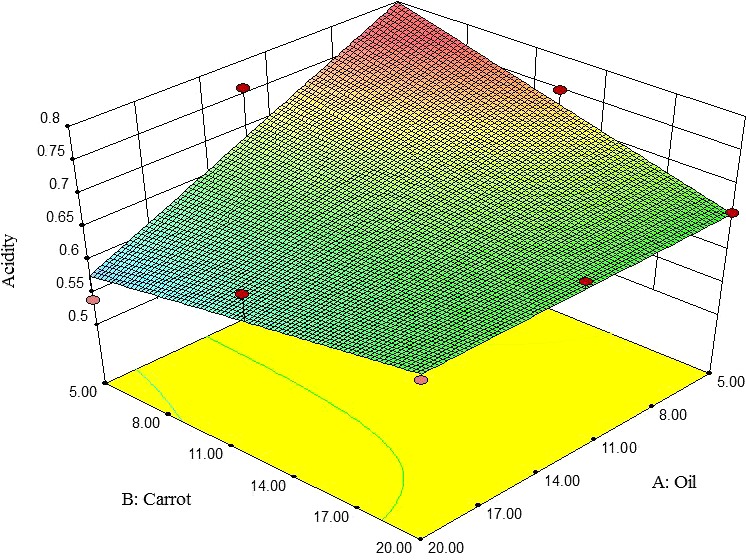
The interactive effect of soybean oil and carrot extract on acidity of pizza cheese

The acidity of pizza cheese samples was decreased by increasing oil, especially at low levels of carrot. On the other hand, the acidity of pizza cheese samples was significantly decreased by increasing carrot at low levels of oil. However, at high levels of oil, the acidity was gently increased (Figure 2). This is in agreement with findings of Katsiari et al. (2000), Katsiari et al. (2002), and Shahab‐Lavasani et al. (2012) about Feta, low‐fat Kefalograviera and UF white cheeses, respectively. Guinee, Feeney, Auty, and Fox (2002) reported that this increasing acidity could be attributed to microbial growth.

#### 3.2.3 Peroxide value

**Figure 3:**
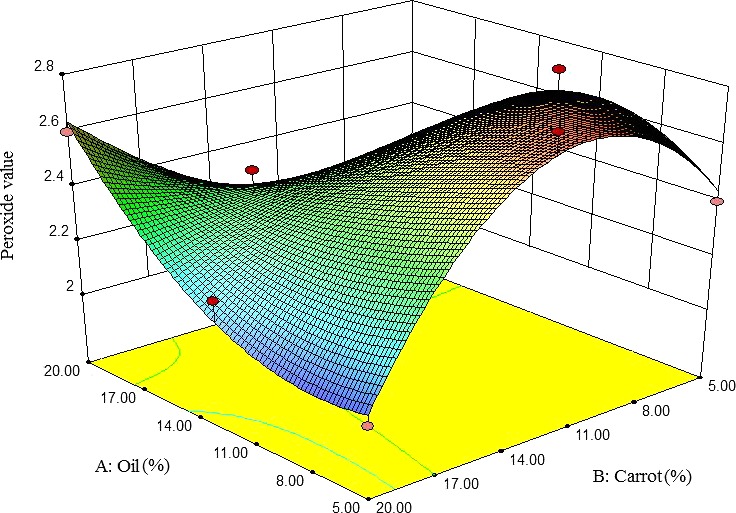
The interactive effect of soybean oil and carrot extract on peroxide value of pizza cheese

The PV of pizza cheese samples was increased by increasing the carrot extract content but subsequently decreased. At high contents of carrot extract, the PV was decreased by decreasing oil content (Figure 3). As is shown in Figure 1c, the lowest PV was observed at 5% oil and 20% carrot extract. Mortensen, Sørensen, and Stapelfeldt (2002) observed that PV of semihard cheeses was increased by increasing the oil content. This result is in agreement with observations of Christensen, Povlsen, and Sørensen (2003) that evaluated processed cheese, using fluorescence spectroscopy and chemometrics during storage.

#### 3.2.4 Color properties

**Figure 4:**
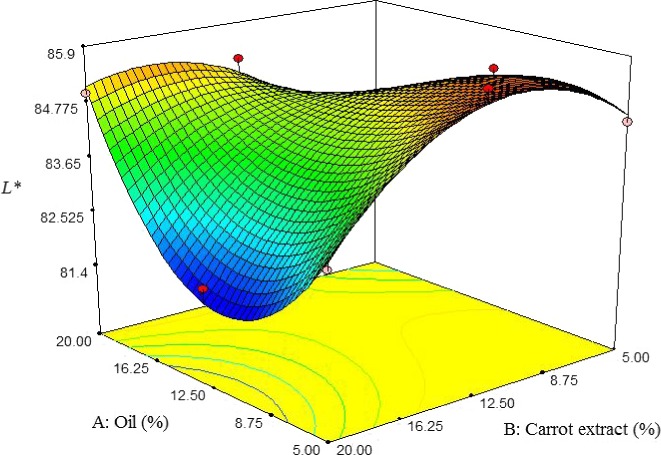
The interactive effect of soybean oil and carrot extract on L* value of pizza cheese

Increasing oil content up to about 10% reduced L* value of pizza cheese samples, but at a higher oil content, the L* value significantly increased. The L* value was gently increased by adding carrot extract; however, at a higher content of carrot extract, the L* value significantly decreased, which is due to high concentrations of carotenoids in the extract, resulting in the opaque color of pizza cheese containing carrot extract (Figure 4) (Dufossé, Mabon, & Binet, 2001). Ghanbari et al. (2012) reported that L* value of Iranian low‐fat white cheese, containing xanthan gum was decreased by increasing the oil content.

**Figure 5:**
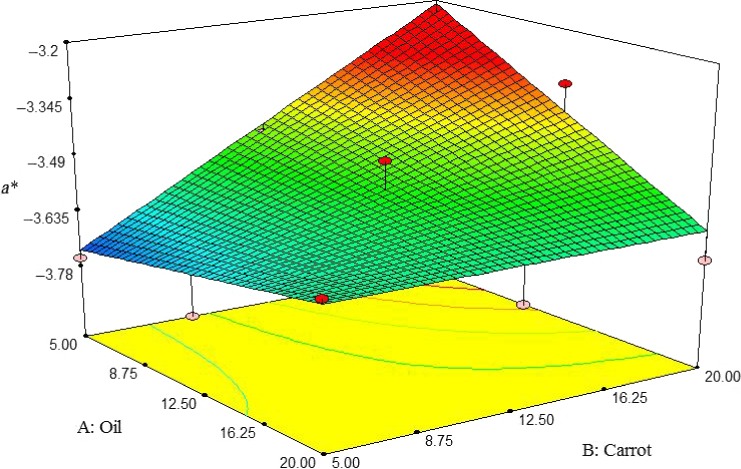
The interactive effect of soybean oil and carrot extract on a* value of pizza cheese

Here, the a*value of pizza cheese containing carrot extract was increased by increasing oil and carrot extract so that the lowest degree of redness was observed at 5% oil and carrot extract content (Figure 5).

**Figure 6:**
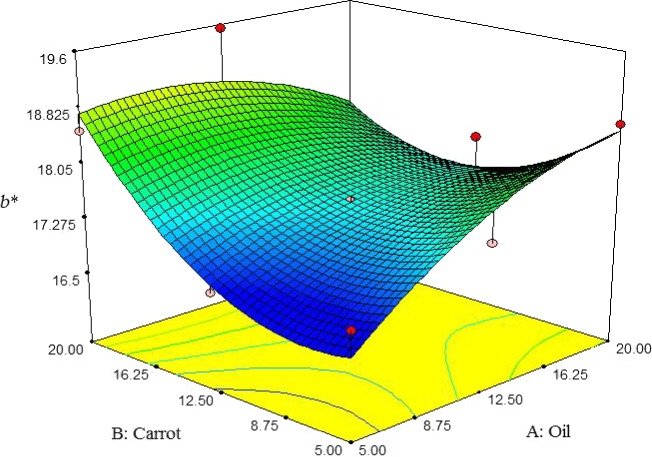
The interactive effect of soybean oil and carrot extract on b* value of pizza cheese

Here, the b* value of pizza cheese samples was increased by increasing the carrot extract content. On the other hand, at low carrot extract content, the b* value was increased by increasing oil content; however, the opposite is true when increasing oil content at high carrot extract content (Figure 6). The highest b* value was observed at 12.5% oil and 20% carrot extract. Similarly, Ghanbari et al. (2012) reported that b* value of cheese was increased by increasing oil content.

#### 3.2.5 Textural properties

##### Cohesiveness

**Figure 7:**
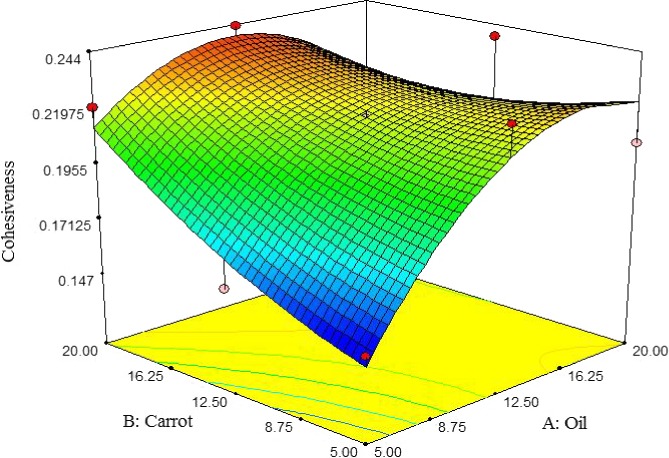
The interactive effect of soybean oil and carrot extract on cohesiveness of pizza cheese

At low content of carrot extract, cohesiveness of pizza cheese samples was initially increased by adding oil up to 9%, but subsequently reduced. On the other hand, the cohesiveness was increased by increasing the carrot extract. The highest cohesiveness pizza cheese containing carrot extract was observed at 5% of oil and carrot extract content (Figure 7). Rashidi, Mazaheri Tehrani, Razavi, and Ghods Rohany (2011) reported that cohesiveness of UF‐Feta Cheese was increased by increasing oil content. Zisu and Shah (2005) demonstrated that cheeses containing a high moisture content (low oil content) have weak internal linkages resulting in softer texture. Beigomi et al. (2013) reported that cohesiveness of cheese based on fungal rennet was increased during storage time; however, cohesiveness of cheese based on plant rennet was increased by the 20th day but subsequently decreased.

##### Gumminess

**Figure 8:**
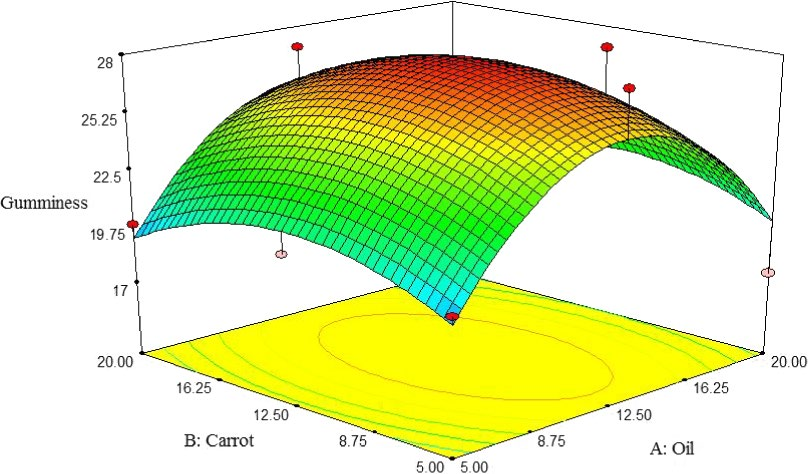
The interactive effect of soybean oil and carrot extract on gumminess of pizza cheese

Figure 8 shows the interactive effect between oil and carrot extract on gumminess of pizza cheese containing carrot extract. The gumminess was initially increased by adding oil and carrot extract up to about 16% and 12.5%, respectively; but it decreased at higher content. So, the lowest gumminess was observed at about 20% oil and 20% carrot extract content. The result of this study is in agreement with the findings of Koca and Metin (2004) for low‐fat Kashar cheese. They reported that gumminess low‐fat fresh kashar cheese was increased by decreasing the oil content. In addition to oil content, moisture content, and protein to moisture ratio has an effect on textural properties of cheeses (Koca & Metin, 2004).

##### Chewiness

**Figure 9:**
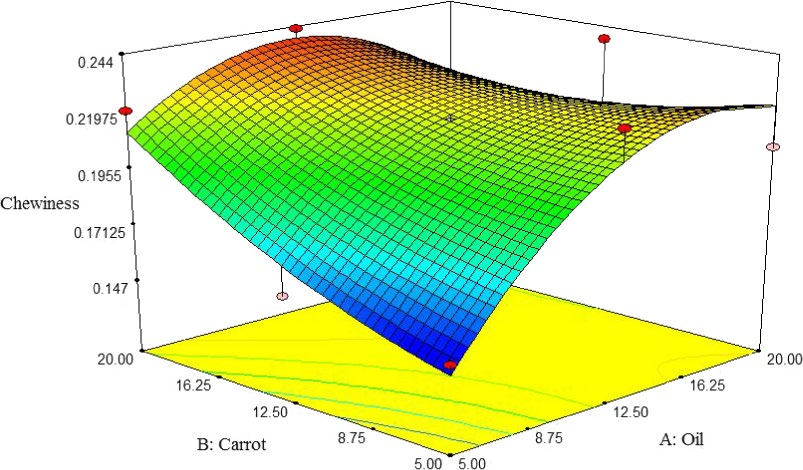
The interactive effect of soybean oil and carrot extract on chewiness of pizza cheese

The chewiness of pizza cheese containing carrot extract was increased by increasing the oil and carrot extract content. The lowest chewiness of pizza cheese samples was observed at 5% oil and carrot extract (Figure 9). Similarly, Koca and Metin (2004) reported that chewiness of low‐fat Kashar cheese was reduced by decreasing the oil content.

##### Adhesiveness

**Figure 10:**
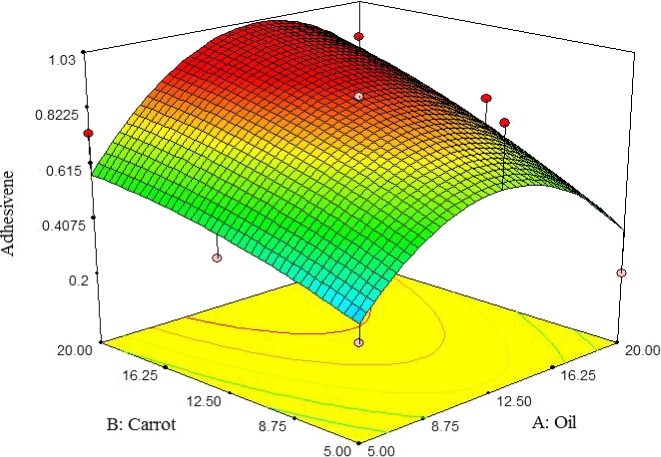
The interactive effect of soybean oil and carrot extract contents on adhesiveness of pizza cheese

The adhesiveness of pizza cheese containing carrot extract was increased by increasing the carrot extract. The adhesiveness was initially increased by adding oil up to 12.5%, but subsequently reduced. The lowest adhesiveness of pizza cheese samples was observed at 5% oil and carrot extract content (Figure 10). Dimitreli and Thomareis (2007) reported that high oil content makes the protein matrix weak, resulting in increased adhesiveness.

##### Springiness

**Figure 11:**
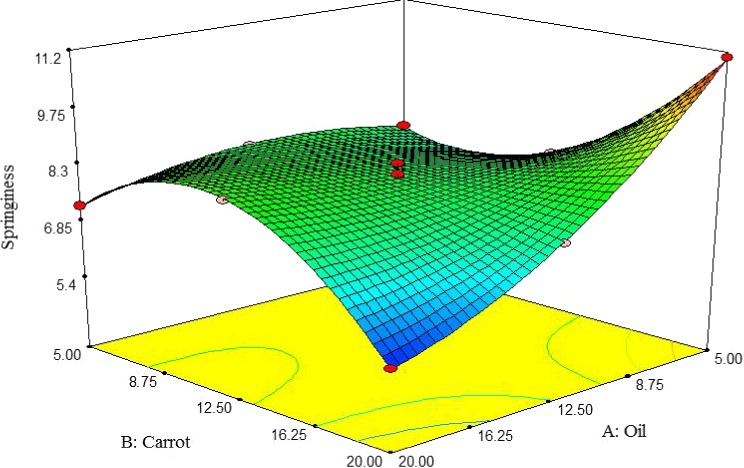
The interactive effect of soybean oil and carrot extract on springiness of pizza cheese

According to Figure 11, the springiness of pizza cheese samples was increased by increasing the carrot extract and decreasing the oil content. The highest springiness of pizza cheese samples was observed at 5% oil and 20% carrot extract (Figure 11). The result of this study is in agreement with the findings of Zisu and Shah (2005).

##### Stretch

**Figure 12:**
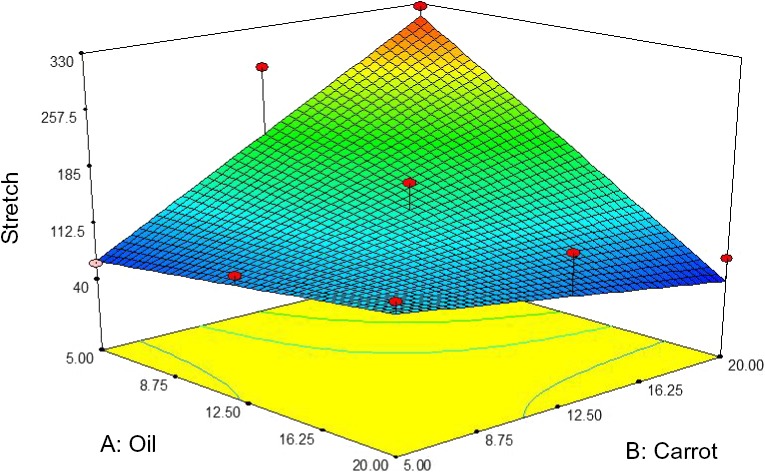
The interactive effect of soybean oil and carrot extract on stretch of pizza cheese

Figure 12 shows the interactive effect between oil and carrot extract on a stretch of pizza cheese containing carrot extract. The stretch was increased by increasing the carrot extract and decreasing the oil content. The highest stretch of pizza cheese samples was observed at 5% oil and 20% carrot extract (Figure 12). Mizuno, R., & Lucey, J. A. (2005) reported that stretch and meltability of nonfat pasta filata cheese was increased by adding trisodium citrate. Fox, Guinee, Cogan, and McSweeney (2000) demonstrated that the stretch of cheese is dependent to the casein‐associated calcium content so that too low or too much of calcium reduces the stretch.

##### Meltability

**Figure 13:**
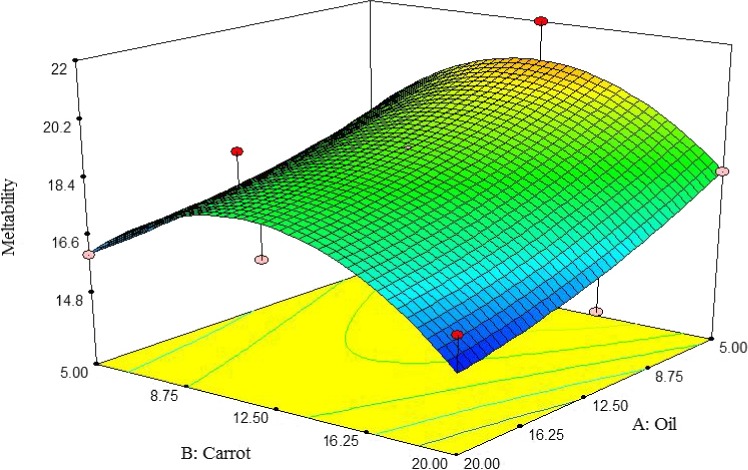
The interactive effect of soybean oil and carrot extract on meltability of pizza cheese

According to Figure 13, meltability of pizza cheese samples was increased by decreasing oil content. On the other hand, the meltability was initially increased by adding carrot extract up to 12.5%, but subsequently decreased. The highest meltability of pizza cheese containing carrot extract was observed at 5% oil and 12.5% carrot extract (Figure 13). The result of this study is in agreement with findings of Zalazar et al. (2002), and Hassan and Abd‐El‐Gawad (2000) for mozzarella cheeses. Shirashoji et al. (2006) reported that meltability of processed cheese was decreased by increasing concentration of trisodium citrate.

#### 3.2.6 Sensorial properties

##### Flavor

**Figure 14:**
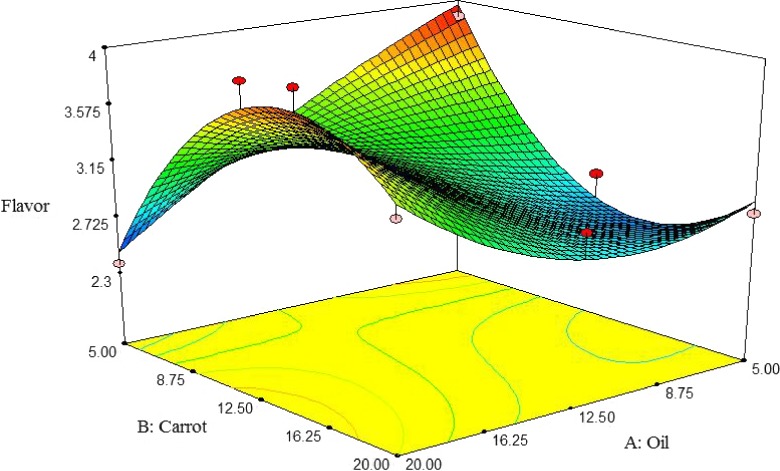
The interactive effect of soybean oil and carrot extract on flavor of pizza cheese

Figure 14 shows the interactive effect between oil and carrot extract on flavor desirability of pizza cheese samples. The flavor desirability was initially increased by adding carrot extract up to 12.5%, but subsequently reduced. The flavor desirability was increased by decreasing oil content. The highest flavor desirability of pizza cheese containing carrot extract was observed at 5% oil and 12.5% carrot extract (Figure 14). Taghvaie, Taslimi, and Mazloumi (2006) reported that partial replacement of milk fat with sunflower oil improved the flavor of cheese. Yu and Hammond (2000) observed the same results for Swiss cheese.

##### Texture

**Figure 15:**
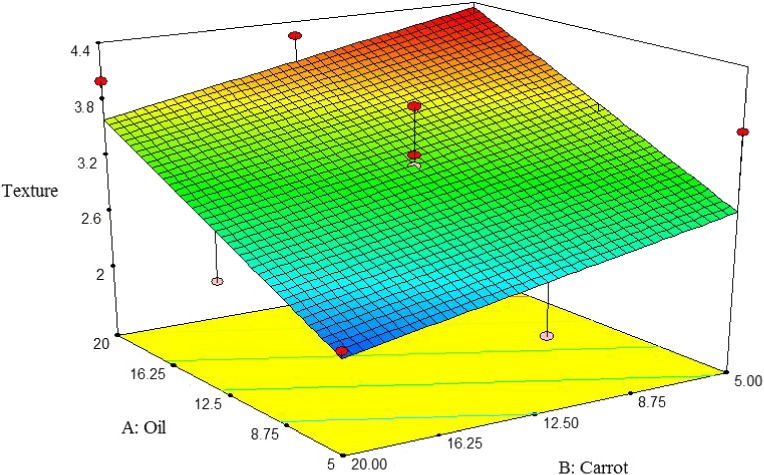
The interactive effect of soybean oil and carrot extract on flavor of pizza cheese

Texture desirability of pizza cheese containing carrot extract was increased by decreasing carrot extract content and increasing the oil content. The highest textural desirability was observed at 5% oil and 20% carrot extract (Figure 15). Similarly, Sipahioglu, Alvarez, and Solano‐Lopez (1999) reported that textural desirability of feta cheese was decreased by increasing fat.

##### Overall acceptability

**Figure 16:**
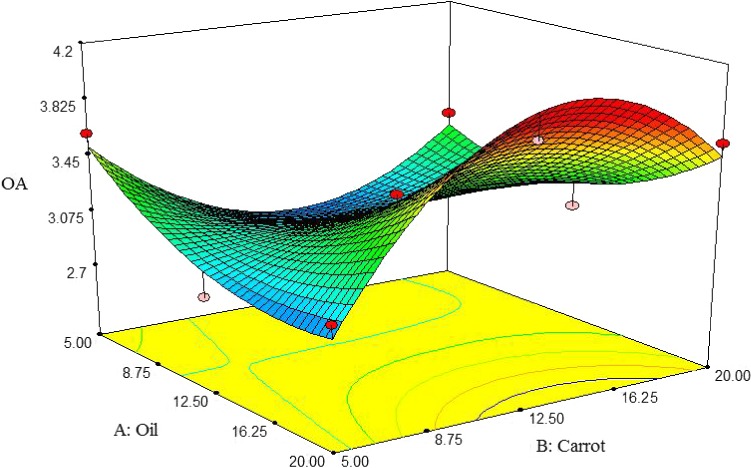
The interactive effect of soybean oil and carrot extract on overall acceptability of pizza cheese

Figure 16 describes the interactive effect between oil and carrot extract on OA of pizza cheese samples. At low carrot extract content, the OA was increased by decreasing oil content; however, the opposite is true when decreasing oil content at high carrot extract content. The OA was initially increased by adding carrot extract up to 12.5%, but subsequently reduced. The highest OA was observed at 5% oil and 12.5% carrot extract (Figure 16).

### 3.3 Optimization

The numerical optimization technique performed to optimize the formulation, when weight and importance value for all of the responses were considered equal (Yolmeh et al., 2017). The PV, L*, a*, b*, meltability, stretch, cohesiveness, springiness, gumminess, chewiness, adhesive force, flavor, texture, and OA attributes were considered for the optimization formulation of pizza cheese. The formulation upon 20% oil and 10.88% carrot extract was found as the optimal formulation for pizza cheese containing carrot extract. The PV, L*, a*, b*, meltability, stretch, cohesiveness, springiness, gumminess, chewiness, adhesive force, flavor, texture, and OA were acquired 2.17, 84.46, −3.61, 17.61, 18.08, 88.73, 0.45, 8.549, 21.99, 0.21, 0.585, 3.66, 4.05, and 3.96, respectively; as the predicted results whose composite desirability values were equal to 0.75. The experimental results of PV, L*, a*, b*, meltability, stretch, cohesiveness, springiness, gumminess, chewiness, adhesive force, flavor, texture, and OA at the optimum formulation were 2.23, 82.51, −3.69, 18.05, 17.86, 85.61, 0.41, 7.874, 23.7, 0.27, 0.61, 3.50, 3.95, and 3.65, respectively.

## 4 CONCLUSIONS

RSM was successfully used for optimizing formulation of pizza cheese containing carrot juice. Results of this study revealed that the linear model was more adequate than other models for moisture and fracture force values of pizza cheese samples; however for acidity, stretch, a*, PV, cohesiveness, flavor, and overall acceptability, the 2FI model was suitable. The quadratic model had more accuracy on L*, b*, hardness, chewiness, gumminess, adhesiveness force, and stiffness of pizza. However, the cubic model was more adequate for meltability and springiness. A formulation upon 20% oil and 10.88% carrot extract was found as the optimal formulation for pizza cheese containing carrot extract. At the optimal formulation, PV, L*, a*, b*, meltability, stretch, cohesiveness, springiness, gumminess, chewiness, adhesive force, flavor, texture, and OA at the optimum formulation were measured 2.23, 82.51, −3.69, 18.05, 17.86, 85.61, 0.41, 7.874, 23.7, 0.27, 0.61, 3.50, 3.95, and 3.65, respectively.
